# Independent association of a 17q21 variant with exacerbations in type 2–low adult asthma

**DOI:** 10.1016/j.jacig.2025.100511

**Published:** 2025-06-10

**Authors:** Yumi Ishiyama, Hisako Matsumoto, Hironobu Sunadome, Yuji Tohda, Takahiko Horiguchi, Hideo Kita, Kazunobu Kuwabara, Keisuke Tomii, Kojiro Otsuka, Masaki Fujimura, Noriyuki Ohkura, Katsuyuki Tomita, Akihito Yokoyama, Hiroshi Ohnishi, Yasutaka Nakano, Tetsuya Oguma, Soichiro Hozawa, Yoshihiro Kanemitsu, Tadao Nagasaki, Isao Ito, Tsuyoshi Oguma, Hideki Inoue, Tomoko Tajiri, Toshiyuki Iwata, Junya Ono, Shoichiro Ohta, Tomomitsu Hirota, Mayumi Tamari, Akio Niimi, Kenji Izuhara, Michiaki Mishima, Toyohiro Hirai, Kinki Hokuriku Airway disease Conference (KiHAC) Study

**Affiliations:** a Department of Respiratory Medicine, Kyoto University Graduate School of Medicine, Kyoto, Japan; b Department of Respiratory Medicine and Allergology, Kindai University Faculty of Medicine, Osaka, Japan; c Kindai University Hospital, Osaka, Japan; d Department of Respiratory Internal Medicine, Fujita Health University Second Educational Hospital, Toyoake, Japan; e Department of Respiratory Medicine, Toyota Regional Medical Center, Toyota, Japan; f Department of Respiratory Medicine, Takatsuki Red Cross Hospital, Osaka, Japan; g Department of Internal Medicine/Respiratory Medicine, Fujita Health University School of Medicine, Nagoya, Japan; h Department of Respiratory Medicine, Kobe City Medical Center General Hospital, Kobe, Japan; i Department of Respiratory Medicine, Shinko Hospital, Kobe, Japan; j Department of Respiratory Medicine, Cellular Transplantation Biology, Kanazawa University Graduate School of Medicine, Kanazawa, Japan; k Department of Respiratory Medicine, National Hospital Organization Nanao Hospital, Nanao, Japan; l Respiratory Medicine, Kanazawa University Graduate School of Medical Sciences, Kanazawa, Japan; m Department of Respiratory Medicine and Allergology, Faculty of Medicine, Kinki University, Osaka, Japan; n Department of Respiratory Medicine, National Hospital Organization Yonago Medical Center, Tottori, Japan; o Department of Respiratory Medicine and Allergology, Kochi Medical School Kochi University, Kochi, Japan; p Division of Respiratory Medicine, Department of Internal Medicine, Shiga University of Medical Science, Otsu, Japan; q Oguma Family Clinic, Kusatsu, Japan; r Department of Respiratory Medicine, Hiroshima Allergy and Respiratory Clinic, Hiroshima, Japan; s Department of Respiratory Medicine, Allergy and Clinical Immunology, Nagoya City University School of Medical Sciences, Nagoya, Japan; t Department of Respiratory Medicine and Allergology, Kindai University Nara Hospital, Nara, Japan; u Department of Respiratory Medicine, Kyoto City Hospital, Kyoto, Japan; v Asthma & Lung Clinic Tokyo, Tokyo, Japan; w Department of Respiratory Medicine, Kyoto Katsura Hospital, Kyoto, Japan; x Shino-Test Corp, Sagamihara, Japan; y Conolab, Inc, Kanagawa, Japan; z Department of Laboratory Medicine, Saga Medical School, Saga, Japan; aa Department of Pharmaceutical Sciences, School of Pharmacy at Fukuoka, International University of Health and Welfare, Okawa, Japan; bb Division of Molecular Genetics, Research Center for Medical Science, The Jikei University School of Medicine, Tokyo, Japan; cc Division of Allergy, Department of Biomolecular Sciences, Saga Medical School, Saga, Japan; dd Department of Respiratory Medicine, Saiseikai-Noe Hospital, Osaka, Japan

**Keywords:** Type 2–low asthma, asthma exacerbation, 17q21, *GSDMB*, *ORMDL3*, rs7216389

## Abstract

**Background:**

The genetic factors contributing to exacerbations in type 2–low asthma are not well understood.

**Objective:**

We sought to clarify the association between variants in gasdermin B/orosomucoid-like 3 (*GSDMB/ORMDL3*) on 17q21 and exacerbations in type 2–low asthma.

**Methods:**

This follow-up study of the multicenter Kinki Hokuriku Airway disease Conference (KiHAC) enrolled adults with asthma who were receiving inhaled corticosteroids. It examined associations between asthma exacerbations requiring systemic corticosteroids over 2 years and clinical and genetic factors in patients with the type 2–low endo-genotype, defined by serum periostin levels lower than 95 ng/mL and the *IL4RA* rs8832 A allele. Exacerbation risks were also evaluated in patients with the type 2–low genotype, defined by both the *POSTN* rs3829365 C allele and the *IL4RA* rs8832 A allele, using the KiHAC and replication cohorts. The genetic variant rs7216389 in *GSDMB* was the primary focus for assessing genetic risk.

**Results:**

A total of 115 patients with the type 2–low endo-genotype were analyzed (mean age, 62 years; 76.5% female). During the 2-year follow-up, 32 patients experienced 1 or more exacerbation. Multivariate analysis identified the rs7216389 TT genotype, recent exacerbations, female sex, and higher body mass index as independent risk factors for asthma exacerbations in patients with the type 2–low endo-genotype. The association between the rs7216389 TT genotype and exacerbations was confirmed in patients with the type 2–low genotype in the KiHAC (n = 89) and replication (n = 125) cohorts.

**Conclusions:**

The rs7216389 TT variant on 17q21 may be an independent risk factor for exacerbations in adults with type 2–low asthma, highlighting the role of *GSDMB* in its pathophysiology.

## Graphical abstract

**Figure:**
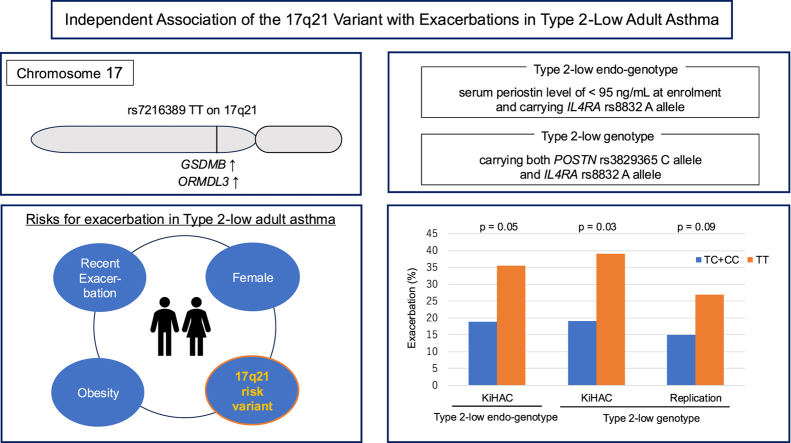

Asthma is commonly classified into type 2–high or type 2–low endotypes, depending on the level of type 2 inflammation.^1^ Although the underlying mechanisms of type 2–high asthma and its exacerbations are now well understood, with effective therapeutic approaches such as biologics, type 2–low asthma still presents significant unmet medical needs. Factors such as obesity, smoking, female sex, and chronic airway infections are known characteristics of type 2–low asthma,^2^^,^^3^ but the genetic risks associated with exacerbations in type 2–low asthma remain underexplored.

Since the first genome-wide association study in 2007,^4^ the 17q21 locus, which includes gasdermin B (*GSDMB*) and orosomucoid-like 3 (*ORMDL3*), has emerged as the most significant susceptibility locus in childhood and early-onset asthma, independent of atopic predisposition.^5^, ^6^, ^7^, ^8^ Although its association with pediatric asthma is well established, this locus has also been reported to be linked to disease severity in adult asthma.^9^^,^^10^

Variants in *GSDMB/ORMDL3* lead to increased expression of GSDMB and ORMDL3 in immune cells^4^^,^^11^^,^^12^ and airway epithelial cells (AECs)^9^ and are linked not only to asthma susceptibility but also to disease severity and exacerbation risks in both children^6^^,^^13^ and adults,^9^^,^^10^ suggesting a broader role in asthma pathophysiology across different age groups.

GSDMB overexpression enhances airway hyperresponsiveness and remodeling without accompanying airway inflammation.^14^ Furthermore, GSDMB promotes pyroptosis, a form of programmed cell death, contributing to airway epithelial damage, particularly during viral infections.^15^^,^^16^ Elevated ORMDL3 levels lead to dysregulated sphingolipid metabolism,^17^ which may increase airway hyperreactivity^18^^,^^19^ in the absence of airway inflammation. In addition, the mechanistic contribution of the 17q21 locus in asthma may be supported by increased expression of other ORMDL family members, that is, ORMDL1 and ORMDL2.^20^ Notably, recent studies have shown a stronger association between the rs7216389 variant and asthma in nonallergic condition compared with allergic condition,^9^^,^^17^^,^^21^ indicating that the 17q21 region, containing *GSDMB*/*ORMDL3*, plays a key role in the pathophysiology of type 2–low asthma, particularly nonallergic asthma.

The aim of this 2-year follow-up cohort study was to evaluate the impact of the rs7216389 variant and clinical factors on exacerbations in type 2–low adult asthma on the basis of the hypothesis that this variant may also play a role in adult asthma. The rs6967330 variant in cadherin-related family member 3 (*CDHR3*), which encodes a receptor for human rhinovirus C, was also analyzed, because studies on *CDHR3* in adults are limited, despite its identification as a risk factor for asthma exacerbation in children^5^^,^^22^^,^^23^ similarly to *GSDMB*/*ORMDL3*.

## Methods

### Study design and patients

This was a multicenter, 2-year follow-up study of the Kinki Hokuriku Airway disease Conference (KiHAC). A total of 217 Japanese adults with asthma, who had been on inhaled corticosteroids for more than 4 years, were recruited and followed up for 2 years. Current and past smokers (>10 pack years or smoking in the year before enrollment) or who had comorbidities of other respiratory diseases were excluded.^24^

Nonatopic asthma was defined as having a total IgE less than 170 IU/mL and no sensitization to common aeroallergens (grass pollen, tree pollen, house dust mites, cat, dog, and a mixture of molds), that is, a specific IgE level less than 0.35 UA/mL. All participants provided informed consent, and the study adhered to the Declaration of Helsinki, with approval from the institutional review boards at each center. The study was registered in the University Hospital Medical Information Network (UMIN) Clinical Trials Registry (Registry ID no. UMIN000002414). A replication cohort was included to validate the findings from the KiHAC cohort.

### Measurements

At enrollment, all participants underwent medical history review, assessment of recent exacerbations, pulmonary function tests, and blood tests. Recent exacerbations were defined as those occurring within 6 months before enrollment. Blood tests included total serum IgE levels, sensitization to common aeroallergens, serum periostin (Shino-Test Corp, Kanagawa, Japan), and genotyping. Details of these measurements were previously reported.^24^ Type 2–low endo-genotype was defined as having a serum periostin level less than 95 ng/mL and carrying the *IL4RA* rs8832 A allele. Patients with the rs8832 GG genotype were excluded, because this genotype is thought to enhance T_H_2 inflammation.^25^, ^26^, ^27^, ^28^ The cutoff for serum periostin of 95 ng/mL was determined on the basis of previous findings indicating that levels higher than this threshold could identify adults with asthma with refractory type 2 inflammation despite appropriate inhaled corticosteroid treatment.^24^ The number of asthma exacerbations, defined as those requiring systemic corticosteroids, was tracked during the 2-year follow-up.

### Genotyping

This study examined the variants rs7216389 and rs4065275 on 17q21, as well as rs6967330 on *CDHR3*, as target variants. In addition, variants rs8832 on *IL4RA* and rs3829365 on *POSTN* were analyzed to stratify patients in both the KiHAC and replication cohorts. Genotyping was conducted using the TaqMan genotyping assay (Applied Biosystems, Tokyo, Japan) following the manufacturer’s instructions. The analysis was performed using the 7300 Real-Time PCR System (Applied Biosystems).

### Replication cohort

The replication cohort included patients with asthma who attended Kyoto University Hospital and were followed up for 1 year. Further details are provided in a previous report.^26^ Asthma exacerbation was defined as the need for systemic corticosteroids, and records of these exacerbation events were collected. All participants were genotyped, and those carrying both the *POSTN* rs3829365 C allele and the *IL4RA* rs8832 A allele were included in the replication cohort as the type 2–low genotype, because this combination was associated with significantly lower serum periostin levels in the KiHAC study compared with other genotypes.^26^ The type 2–low genotype was considered to closely align with the type 2–low endo-genotype, defined as having a serum periostin level less than 95 ng/mL and carrying the *IL4RA* rs8832 A allele.

### Statistical analysis

Statistical analyses were conducted using JMP version 17 (SAS Institute, Tokyo, Japan). Categorical variables were evaluated using the chi-square test or the Fisher exact test, as appropriate. Continuous variables were compared between 2 groups using the Wilcoxon rank-sum test and among 3 or more groups using the Kruskal-Wallis test.

Multivariate logistic regression analysis was performed to assess the risks for future exacerbations. Data are presented as mean ± SD or as median along with the first and third quartiles.

## Results

### Subject demographic characteristics and genotype distributions

Of the 224 patients in the original KiHAC study, 7 were excluded because of loss to follow-up.^26^ Among the remaining 217 subjects, 115 (51%) had serum periostin levels less than 95 ng/mL and carried the rs8832 A allele, indicating they had the type 2–low endo-genotype. This group consisted of 88 female patients, with a mean age of 62.2 ± 13.6 years and a body mass index (BMI) of 23.1 ± 3.4 kg/m^2^. The genotype distributions at CC/CT/TT for rs7216389, AA/AG/GG for rs4065275, and GG/AG/AA for rs6967330 were 6/47/62, 9/43/63, and 97/17/1, respectively. The frequencies of the risk alleles were 0.743 for T at rs7216389, 0.735 for G at rs4065275, and 0.083 for A at rs6967330.

All genotyped data were in Hardy-Weinberg equilibrium. The genotype distributions of rs7216389, rs4065275, and rs6967330 were not significantly different between the 115 patients with type 2–low endo-genotype and the remaining 102 participants from the original KiHAC study.

### Association between rs7216389 TT and asthma exacerbation in type 2–low endo-genotype

**Fig 1:**
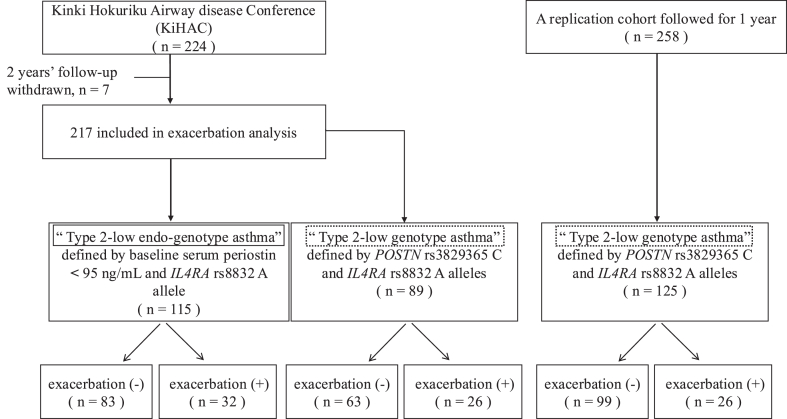
Flowchart of the study.

Among patients with the type 2–low endo-genotype, 32 of 115 (28%) experienced at least 1 exacerbation during the 2-year follow-up (Fig 1). Compared with nonexacerbators, those with exacerbations had a higher proportion of female patients, increased BMI, and more frequent recent exacerbations (Table I). Exacerbators were also more likely than nonexacerbators to receive maintenance oral corticosteroids or biologics (5 of 32 [15.6%] vs 3 of 83 [3.6%]; *P* = .04). The frequency of the rs7216389 TT genotype was higher in exacerbators than in nonexacerbators (68.8% vs 48.2%; *P* = .048). There were no significant differences in the frequencies of variants of rs4065275 or rs6967330 between exacerbators and nonexacerbators (Table I). In multivariate analysis, the rs7216389 TT genotype was identified as a risk factor for future asthma exacerbation, independent of recent exacerbations, female sex, and higher BMI in type 2–low endo-genotype asthma (Table II). The association between rs7216389 TT and asthma exacerbation remained significant even after excluding patients with a history of pediatric asthma (18 exacerbators in 46 with TT [39.1%] vs 9 exacerbators in 46 with CT + CC [19.6%]; *P* = .04). Furthermore, when analyzing only patients with nonatopic asthma in the type 2–low endo-genotype group, the association between rs7216389 TT and exacerbation became more evident (17 exacerbators in 38 with TT [45%] vs 4 exacerbators in 28 with CC + CT [14%]; *P* = .02). This association was not observed in patients with atopic asthma (5 exacerbators in 24 with TT [21%] vs 6 exacerbators in 25 with CC + CT [24%]; *P* = .79).

**Table I:** Characteristics of patients with type 2–low endo-genotype,∗ categorized by the presence of exacerbations† during the 2-y follow-up

Characteristics	Subsequent exacerbations		*P* value
	(−) (n = 83)	(+) (n = 32)	
Sex: female/male, n	58/25	30/2	.007
Age (y)	60.0 ± 14.4	64.4 ± 12.3	.13
Age (y) of asthma onset	41.5 ± 19.1	44.8 ± 17.2	.39
Pediatric asthma history (+/−), n	18/65	5/27	.61
BMI (kg/m^2^)	22.7 ± 3.4	24.2 ± 4.2	.049
Smoking history, never/ex, n	61/22	29/3	.08
Atopic predisposition‡ (+/−), n	61/22	25/7	.61
GERD (+/−), n	11/72	8/24	.13
Sinusitis (+/−), n	22/61	6/26	.39
Recent exacerbation§ (+/−), n	5/78	12/20	<.0001
FEV_1_% predicted	99 ± 20	97 ± 23	.57
Serum IgE (IU/mL)	156 (50-502)	96 (30-300)	.12
Blood eosinophils (/μL)	244 ± 184	248 ± 149	.54
Blood neutrophils (/μL)	3602 ± 1185	4236 ± 1947	.10
rs7216389, CC + CT/TT, n	43/40	10/22	.048
rs4065275, AA + AG/GG, n	40/43	12/20	.30
rs6967330, GG/AG + AA, n	70/13	27/5	1.00

Data are presented as of enrollment, unless noted otherwise. Data are presented as mean ± SD, median (interquartile range), or counts as appropriate.

*GERD*, Gastroesophageal reflux disease.

∗ Defined by a serum periostin level of <95 ng/mL and the presence of the rs8832 A allele.

† Asthma exacerbations that required systemic corticosteroids.

‡ Considered atopic if any specific IgE antibodies against common inhaled allergens were positive.

§ Presence of exacerbations requiring systemic corticosteroids in the 6 mo before enrollment.

**Table II:** Logistic regression analysis assessing the risk of asthma exacerbations in patients with type 2–low endo-genotype asthma∗ during the 2-y follow-up

Factors	Multivariate analysis, OR (95% CI)	*P* value
*Model 1*		
rs7216389 TT	2.87 (1.05-7.08)	.03
Recent exacerbations†	10.47 (2.96-37.00)	.0003
Sex: female	6.09 (1.22-30.50)	.03
	*R*^2^ = 0.21	
*Model 2*		
rs7216389 TT	3.49 (1.25-9.79)	.02
Recent exacerbations†	12.68 (3.56-45.09)	<.0001
BMI (kg/m^2^)	1.17 (1.03-1.33)	.02
*R*^2^ = 0.20		

*OR*, Odds ratio.

∗ Defined by a serum periostin level <95 ng/mL and the presence of the rs8832 A allele.

† The occurrence of exacerbations that required systemic corticosteroids in the 6 mo before enrollment.

### Association between rs7216389 TT and asthma exacerbation in type 2–low genotype

To evaluate the effect of rs7216389 on exacerbation, the relationship between this variant and exacerbations was analyzed in patients with the type 2–low genotype, defined as those carrying both the *POSTN* rs3829365 C allele and the *IL4RA* rs8832 A allele in the KiHAC cohort (n = 89) and the replication cohort (n = 125). In the replication cohort, 55.2% of participants were female, with a mean age of 64 years (Table III). The type 2–low genotype in the KiHAC cohort included 25 patients with serum periostin levels higher than 95 ng/mL, who were not part of the type 2–low endo-genotype study.

**Table III:** Characteristics of patients with type 2–low genotype∗ in the replication cohort, categorized by the presence of exacerbations† during the 1-y follow-up

Characteristics	Subsequent exacerbations		*P* value
	(−) (n = 99)	(+) (n = 26)	
Sex: female/male, n	54/45	15/11	.77
Age (y)	57.9 ± 15.2	63.5 ± 15.9	.04
BMI (kg/m^2^)	23.2 ± 3.5	21.8 ± 3.2	.09
Atopic predisposition‡ (+/−), n	56/42	14/12	.76
FEV_1_% predicted	102.9 ± 21.5	94.1 ± 26.3	.16
Serum IgE (IU/mL)	105 (47-395)	113 (23-500)	.87
Blood eosinophils (/μL)	314 ± 379	264 ± 214	.71
Maintenance oral corticosteroid or biologics (+/−), n	4/94	3/23	.16
rs7216389, CC+CT/TT, n	53/46	9/17	.09
rs4065275, AA+AG/GG, n	51/48	11/15	.40

Data are presented as of enrollment, unless noted otherwise. Data are presented as mean ± SD, median (interquartile range), or counts as appropriate. Data on atopic predisposition, blood eosinophils, and maintenance oral corticosteroid or biologics were missing for 1 individual.

∗ Defined by carrying both the *POSTN* rs3829365 C allele and the *IL4RA* rs8832 A allele.

† Asthma exacerbations that required systemic corticosteroids.

‡ Considered atopic if any specific IgE antibodies against common inhaled allergens were positive.

Compared with patients with rs7216389 CT + CC, those with the TT genotype had a significantly higher likelihood of experiencing exacerbations (39.1% for TT vs 18.6% for CT + CC; *P* = .03) in the KiHAC type 2–low genotype group. A similar trend was observed in the replication cohort (27.0% for TT vs 14.5% for CT + CC; *P* = .09).

**Fig 2:**
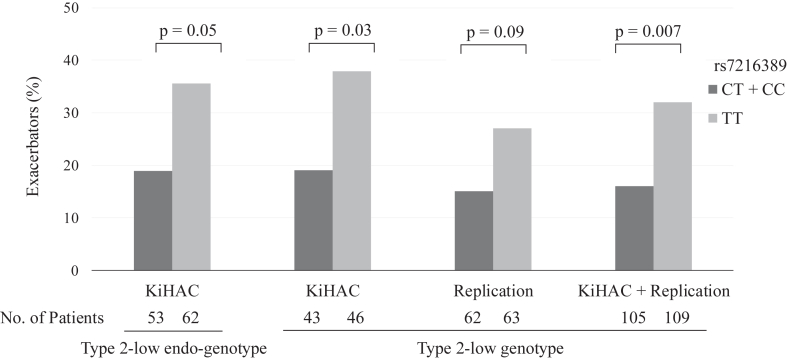
Proportion of exacerbators during the follow-up on the basis of the rs7216389 genotype. The type 2–low endo-genotype was defined by a serum periostin level less than 95 ng/mL and the presence of the *IL4RA* rs8832 A allele. The type 2–low genotype was defined by carrying both the *POSTN* rs3829365 C allele and the *IL4RA* rs8832 A allele.

When combining the KiHAC type 2–low genotype population with the replication cohort, the TT genotype was significantly associated with exacerbations compared with the CT + CC genotypes (32.1% for TT vs 16.2% for CT + CC; *P* = .007) (Fig 2).

When analyzing patients with nonatopic asthma in the type 2–low genotype group, a similar trend was observed in both the KiHAC and replication cohorts (*P* = .08 and *P* = .06, respectively), whereas this trend was not observed in patients with atopic asthma.

### Clinical characteristics of patients with rs7216389 TT

Finally, the characteristics of patients with the rs7216389 TT genotype in type 2–low asthma were analyzed. In the type 2–low endo-genotype group (n = 115), multivariate analysis indicated that the rs7216389 TT genotype was significantly associated with a history of pediatric asthma, lower blood eosinophil counts, and higher FEV1% (Table IV). However, it was not associated with atopic predisposition or elevated serum IgE levels (Table IV). The proportions of patients receiving maintenance oral corticosteroids or biologics were similar between the rs7216389 TT and CT + CC groups (Table IV).

**Table IV:** Association of the rs7216389 genotype with clinical and genetic factors in patients with type 2–low endo-genotype asthma∗

Factors	TT (n = 62)	CT + CC (n = 53)	*P* value
*Univariate analysis*			
Sex: female/male, n	49/13	39/14	.49
Age (y)	62.5 ± 13.3	59.8 ± 14.7	.32
Pediatric asthma history (+/−), n	16/46	7/46	.09
BMI > 25 kg/m^2^ (+/−), n	20/42	15/38	.65
Smoking history, ex/never, n	11/51	14/39	.26
GERD (+/−), n	14/48	5/48	.06
Recent exacerbation (+/−), n	9/53	8/45	.93
FEV_1_% predicted	103.7 ± 20.8	92.1 ± 19.5	.003
Serum total IgE (IU/mL)	99 (31.1-409)	176 (63.5-394.5)	.17
Atopic predisposition (+/−), n	47/15	39/14	.78
Blood neutrophils (/μL)	3853 ± 1616	3692 ± 1255	.84
Blood eosinophils (/μL)	200 ± 150	298 ± 188	.003
Serum periostin (ng/mL)	70.3 ± 16.5	72.4 ± 15.6	.48
Maintenance oral corticosteroid or biologics (+/−), n	4/58	4/49	1.00
rs6967330, GG/AG + AA, n	52/10	45/8	.88
rs4065275, GG/GA + AA, n	55/7	8/45	<.0001

Factors	**Adjusted OR (95% CI)**†		***P* value**
*Multivariate analysis*			
Pediatric asthma history	4.36 (1.35-14.13)		.01
GERD	2.00 (0.60-6.64)		.26
FEV_1_% predicted	1.03 (1.01-1.05)		.005
Blood eosinophils (/μL)	0.997 (0.994-0.999)		.02
*R*^2^ = 0.17			

*GERD*, Gastroesophageal reflux disease; *OR*, odds ratio.

∗ Defined by a serum periostin level of <95 ng/mL and the presence of the rs8832 A allele.

† Corrected for age and sex in the analysis.

The association with blood eosinophil counts was not observed in the type 2–low genotype, in either the KiHAC cohort (n = 89) or the replication cohort (n = 125) (Table V). Finally, no association between the rs7216389 TT genotype and exacerbations was observed when examining patients with low blood eosinophil counts (<150/μL) in both the KiHAC and replication cohorts (Table VI).

**Table V:** Association of the rs7216389 genotype with blood eosinophils in patients (/μL) with type 2–low genotype∗

Cohort	TT	CT + CC	*P* value
KiHAC cohort (n = 89)	237 ± 289	276 ± 299	.39
Replication cohort (n = 124)	317 ± 427	290 ± 252	.99

Data on blood eosinophil counts were missing for 1 patient with type 2–low genotype asthma in the replication cohort.

∗ Defined by carrying both the *POSTN* rs3829365 C allele and the *IL4RA* rs8832 A allele.

**Table VI:** Association of the rs7216389 genotype with exacerbation in patients with low blood eosinophil counts (<150 /μL)

Cohort	TT	CT + CC	*P* value
Exacerbation (+/−), n			
KiHAC cohort (n = 73)	11/34	5/23	.51
Replication cohort (n = 87)	11/32	10/34	.76

Data on blood eosinophil counts were missing for 5 patients with low blood eosinophil counts (<150 /μL) in the replication cohort.

## Discussion

This study is the first to demonstrate that the rs7216389 variant on 17q21 is a risk factor for asthma exacerbation in adults with type 2–low asthma. The association between the rs7216389 TT genotype and asthma exacerbation was identified in the type 2–low endo-genotype, defined by serum periostin levels less than 95 ng/mL and the presence of the rs8832 A allele on *IL4RA*, independent of other known risk factors such as female sex, higher BMI, and recent exacerbations. This association was further validated in the type 2–low genotype defined by the presence of both the rs3829365 C allele on *POSTN*, which encodes periostin, and the rs8832 A allele, in combined populations of the KiHAC and replication cohorts. Moreover, this association was observed only in patients with nonatopic asthma in the type 2–low endo-genotype and type 2–low genotype groups, whereas it was not observed in patients with atopic asthma.

Since the identification of 17q21 as a susceptible locus for childhood asthma in the genome-wide association study in 2007,^4^ this association has been consistently replicated in various ethnic populations.^7^ Risk variants in this locus, particularly rs7216389, are linked not only to asthma susceptibility but also to its severity and exacerbations in both children^6^^,^^13^ and adults.^9^^,^^10^ In addition, these variants are associated with increased expression of *GSDMB*/*ORMDL3* in immune cells^4^^,^^11^^,^^12^ and AECs.^9^ These findings indicate that the increased expression of *GSDMB*/*ORMDL3* because of the 17q21 risk variant affects the pathophysiology of asthma, regardless of the age of onset. Our study builds on this knowledge by demonstrating that the rs7216389 variant independently increases the risk of exacerbation in type 2–low adult asthma, observed in both the type 2–low endo-genotype and the type 2–low genotype.

Some studies have identified associations between the overexpression of *GSDMB/ORMDL3* or their variants and type 2 inflammation.^12^^,^^29^ However, our previous study found no association between rs7216389 TT and exacerbations in type 2–high asthma (defined by serum periostin ≥95 ng/mL).^26^ Furthermore, higher *GSDMB* expression correlates with an increased number of exacerbations and with genes related to interferon signaling and the T_H_1 pathway in adult asthma.^9^ Higher *GSDMB* levels are also associated with reduced type 2 immunity in nasal brushings from children, regardless of their asthma status.^21^ These results imply an association between *GSDMB* expression and type 1 inflammation rather than a direct link to type 2 inflammation. Taken together, these findings suggest that type 2 inflammation may not be essential for the relationship between rs7216389 in *GSDMB/ORMDL3* and asthma exacerbations.

An increase in GSDMB^14^ or ORMDL3^30^ is linked to airway hyperresponsiveness and remodeling in the absence of airway inflammation. GSDMB is expressed in AECs and promotes pyroptosis, particularly during viral infections and IFN-γ stimulation.^15^^,^^16^

Recently, Jakwerth et al^21^ found that both the rs7216389 risk allele (T) and elevated *GSDMB* expression were associated with a shift from type 1/3 interferons to IFN-γ expression as well as killer cell–dependent cell lysis signature in nasal brush samples from children. They suggested that the combination of increased pyroptosis and killer cell–dependent cell lysis might disrupt the airway epithelial barrier in individuals with risk variants on 17q21, leading to a higher susceptibility to infections, suppressed type 1/3 interferon expression, and subsequent exacerbations. The association between increased *GSDMB* expression and the interferon signature was also evident in type 1 immunity.^21^

The involvement of *GSDMB/ORMDL3* in type 2–low asthma is further illustrated by the effect of *ORMDL3* on sphingolipid dysregulation. Although the specific roles of sphingolipids are not well understood, disruptions in sphingolipid synthesis are recognized as a risk factor for asthma.^7^ Overexpression of *ORMDL3* reduces *de novo* sphingolipid synthesis and is associated with risk variants on 17q21, including rs7216389, in a manner dependent on the risk allele.^17^ This reduction is more pronounced in the low blood eosinophil group (<300/μL) compared with the high blood eosinophil group (>300/μL) in childhood asthma.^17^

It remains uncertain whether GSDMB or ORMDL3 plays a critical role in exacerbations in patients with type 2–low asthma, although the role of GSDMB has received more attention recently.^21^ In this study, rs4065275, another risk variant on 17q21 that is in strong linkage disequilibrium with rs7216389,^12^ was not associated with exacerbations. rs4065275 is located in the intron of *ORMDL3*, whereas rs7216389 is located in the intron of *GSDMB*. An expression quantitative trait locus analysis of AECs from adults with asthma indicated that rs7216389 is more likely to contribute to increased GSDMB expression than rs4065275.^9^ This may explain why rs7216389, rather than rs4065275, was associated with exacerbations in this study, suggesting a greater involvement of GSDMB than ORMDL3 in exacerbations in type 2–low asthma.

Woodruff et al^31^ demonstrated that T_H_2-high and T_H_2-low asthma phenotypes, currently often referred to as type 2–high and type 2–low asthma phenotypes, can be differentiated on the basis of the expression levels of IL-13– and IL-13–inducible genes, including *POSTN*, which encodes periostin. As a result, this study established 2 definitions of “type 2–low asthma” using serum periostin and *IL4RA* for endo-genotyping and *POSTN* and *IL4RA* for genotyping. Although it is certainly true that the presence of the *POSTN* rs3829365 C allele does not necessarily correspond to low serum periostin levels, the analysis of the type 2–low genotype population supported the findings of the type 2–low endo-genotype. In contrast, no association was found between the rs7216389 TT genotype and exacerbations in patients with low blood eosinophil counts (<150/μL). The reasons for the discrepancy between type 2–low defined by low serum periostin and its related genetic polymorphisms and type 2–low defined by low blood eosinophil counts are not fully understood. However, it is possible that IL-13/IL-4–low and eosinophil-low conditions are not identical. Future studies should aim to identify genotypic variants associated with type 2–low asthma.

Carrying the rs7216389 TT genotype was associated with a history of pediatric asthma but not with atopic predisposition or total serum IgE levels in patients with the type 2–low endo-genotype in the KiHAC study, aligning with previous studies.^4^^,^^6^^,^^7^^,^^11^^,^^13^^,^^32^ However, the significant association between rs7216389 TT and asthma exacerbation remained after excluding patients with a history of pediatric asthma from the analysis.

The small sample size prevented a comparison of the strength of this association between patients with pediatric asthma and those with adult-onset asthma. In the type 2–low endo-genotype and genotype groups, the association between the rs7216389 TT variant and asthma exacerbations was observed in patients with nonatopic asthma but not in those with atopic asthma. This may support the idea that the risk variant on 17q21, rs7216389 TT, is associated with type 2–low asthma.

Variants of rs6967330 on *CDHR3*, a receptor for human rhinovirus C, are recognized as risk factors for severe exacerbations in childhood asthma.^5^^,^^22^^,^^23^ However, this study found no association between the rs6967330 variant and exacerbations in type 2–low adult asthma. Kanazawa et al^33^ noted that the association between the *CDHR3* variant and early-onset asthma was stronger in atopic individuals compared with nonatopic individuals.^33^ The effect of the *CDHR3* variant may be more pronounced in patients with allergic asthma, because plasmacytoid dendritic cells in these individuals have a reduced ability to produce high levels of IFN-α/β in response to viruses.^34^

This study has several limitations. First, serum periostin levels were not measured in the replication cohort; instead, we relied on the type 2–low genotype, which has previously been shown to have significantly lower serum periostin levels than other genotype combinations. Second, the triggers of exacerbations were not evaluated. The effects of variants on 17q21 and *CDHR3* may be more pronounced in exacerbations specifically caused by viral infections. Lastly, the sample size was relatively small, suggesting that future studies with larger cohorts are needed.

Carrying the rs7216389 TT variant on 17q21 may be an independent risk factor for exacerbation in type 2–low adult asthma. Further studies are needed to clarify the mechanisms underlying exacerbations in type 2–low asthma and to identify potential therapeutic targets related to GSDMB.

**Clinical implications**

The rs7216389 TT variant on 17q21, which increases *GSDMB* and *ORMDL3* expression, may independently increase the risk of exacerbations in adults with type 2–low asthma, highlighting their roles in its pathophysiology.

## Disclosure statement

This study was funded by the Kinki Hokuriku Airway disease Conference, the Adaptable and Seamless Technology Transfer Program through target-driven R&D, Japan Science and Technology Agency, Grants-in-Aid for Scientific Research, and the Japan Society for the Promotion of Science.

Disclosure of potential conflict of interest: H. Sunadome reports royalties from Philips Japan, Fukuda Denshi, Fukuda Lifetec Keiji, and ResMed. Y. Tohda reports grants from 10.13039/100019271Kyorin Pharmaceutical, 10.13039/100017346Nippon Boehringer Ingelheim, and Taiho Pharmaceutical; and lecture fees from AstraZeneca, Kyorin Pharmaceutical, and GlaxoSmithKline. H. Kita reports lecture fees from AstraZeneca and GlaxoSmithKline. A. Yokoyama reports lecture fees from GlaxoSmithKline, Sanofi, and Nippon Boehringer Ingelheim. H. Matsumoto and H. Ohnishi report lecture fees from AstraZeneca, GlaxoSmithKline, Kyorin Pharmaceutical, Novartis Pharma, and Sanofi. S. Hozawa reports lecture fees from AstraZeneca, GlaxoSmithKline, Novartis Pharmaceuticals, and Kyorin Pharmaceutical. Y. Kanemitsu reports grant from MSD Life Foundation and MSD; lecture fees from GlaxoSmithKline, AstraZeneca, Kyorin Pharmaceutical, Novartis Pharma, Sanofi, and Zeria; and support for attending meetings from GlaxoSmithKline and Sanofi. T. Oguma reports lecture fees from Kyowa Kirin, AstraZeneca, GlaxoSmithKline, and Mitsubishi Tanabe Pharma. J. Ono is the CEO of Conolab, Inc, but the company has no relation to this study. A. Niimi reports lecture fees from GlaxoSmithKline, AstraZeneca, Kyorin Pharmaceutical, Novartis Pharma, and Sanofi. K. Izuhara reports grant and lecture fees from Shino-Test Corp. T. Hirota reports lecture fees from AstraZeneca and Sanofi. The rest of the authors declare that they have no relevant conflicts of interest.
